# Head circumferences of children born to HIV-infected and HIV-uninfected mothers in Zimbabwe during the preantiretroviral therapy era

**DOI:** 10.1097/QAD.0000000000001196

**Published:** 2016-09-07

**Authors:** Ceri Evans, Bernard Chasekwa, Robert Ntozini, Jean H. Humphrey, Andrew J. Prendergast

**Affiliations:** a Zvitambo Institute for Maternal and Child Health Research, Harare, Zimbabwe; b Blizard Institute, Queen Mary University of London, London, UK; c Department of International Health, Johns Hopkins Bloomberg School of Public Health, Baltimore, Maryland, USA.

**Keywords:** Africa, children, head circumference, HIV, microcephaly

## Abstract

**Objectives:**

To describe the head growth of children according to maternal and child HIV infection status.

**Design:**

Longitudinal analysis of head circumference data from 13 647 children followed from birth in the ZVITAMBO trial, undertaken in Harare, Zimbabwe, between 1997 and 2001, prior to availability of antiretroviral therapy (ART) or cotrimoxazole prophylaxis.

**Methods:**

Head circumference was measured at birth, then at regular intervals through 24 months of age. Mean head circumference-for-age *Z*-scores (HCZ) and prevalence of microcephaly (HCZ < −2) were compared between HIV-unexposed children, HIV-exposed uninfected (HEU) children and children infected with HIV *in utero* (IU), intrapartum (IP) and postnatally (PN).

**Results:**

Children infected with HIV *in utero* had head growth restriction at birth. Head circumference *Z*-scores remained low throughout follow-up in IP children, whereas they progressively declined in IU children. During the second year of life, HCZ in the PN group declined, reaching a similar mean as IP-infected children by 21 months of age. Microcephaly was more common among IU and IP children than HIV-uninfected children through 24 months. HEU children had significantly lower head circumferences than HIV-unexposed children through 12 months.

**Conclusion:**

HIV-infected children had lower head circumferences and more microcephaly than HIV-uninfected children. Timing of HIV acquisition; influenced HCZ, with those infected before birth having particularly poor head growth. HEU children had poorer head growth until 12 months of age. Correlations between head growth and neurodevelopment in the context of maternal/infant HIV infection, and further studies from the current ART era, will help determine the predictive value of routine head circumference measurement.

## Introduction

Children infected with HIV before or around the time of birth have faster disease progression than those infected through breastfeeding [1]. Despite avoiding HIV infection, HIV-exposed uninfected (HEU) children have higher mortality, morbidity and growth failure than HIV-unexposed children [1–3]. HIV-infected children have poorer developmental outcomes than HIV-uninfected children, particularly when antiretroviral therapy (ART) is not initiated early [4].

In infancy, head growth is correlated with brain size [5] and is easily measured. In high HIV prevalence populations, microcephaly may predict poor neurodevelopmental outcomes [6] and is a sign of HIV encephalopathy [7]. Here, we report head growth among children followed from birth in Zimbabwe.

## Methods

Between 1997 and 2000, 14 110 mother–infant pairs were recruited to a randomized trial of maternal/neonatal vitamin A supplementation in Zimbabwe, as previously described [8,9]. Exclusion criteria included multiple pregnancy, birthweight less than 1500 g, maternal plans to leave Harare and life-threatening conditions in the mother/child. ART and cotrimoxazole were not available in public-sector health care at the time.

### Ascertainment of maternal HIV status

All mothers were tested for HIV at baseline using enzyme-linked immunosorbent assay (ELISA) and western blotting [8]. Mothers with negative HIV tests were retested at subsequent visits. Mothers with positive HIV tests were retested at the next visit to confirm status.

### Ascertainment of infant HIV status

Cell pellets and plasma from HIV-exposed children were prepared from whole blood and stored at −70 °C. The last available sample was tested for HIV by ELISA on plasma if aged at least 18 months (GeneScreen; Sanofi Diagnostics Pasteur, Johannesburg, South Africa) or DNA PCR on cell pellets if aged less than 18 months (Roche Amplicor version 1.5; Roche Diagnostic Systems, Alameda, California, USA). If the last sample was positive, earlier samples were tested to determine timing of infection [8].

### HIV infection/exposure status

Children were classified into five categories, as previously described [1,3]:

1. HIV-unexposed: mother HIV-negative throughout study period.
2. HEU: mother HIV-positive at baseline; last available child sample HIV-negative.
3. *In utero*-infected (IU): child HIV PCR-positive at baseline.
4. Intrapartum-infected (IP): child HIV PCR-negative at baseline and HIV PCR-positive at age 6 weeks.
5. Postnatally-infected (PN): child HIV PCR-negative at 6 weeks and HIV-positive after 6 weeks.

Children of mothers who seroconverted during follow-up were excluded. A time-varying analysis was used to categorize children, with those acquiring HIV intrapartum/postnatally categorized as HEU until their final HIV-negative test. To ensure that the HEU group did not become contaminated with IP/PN infants who died or became lost to follow-up before a positive HIV test could be obtained, infants were censored at their last negative HIV test.

### Anthropometric data

Head circumference was measured at each study visit using methods described by Gibson [10]. Measurements were converted into head circumference-for-age *Z*-scores (HCZ) using WHO international standards [11].

### Statistical analyses

Patterns of head circumference from birth to 24 months were described by plotting mean HCZ against age. Unpaired *t* tests were used to compare mean HCZ between groups. Odds ratios for microcephaly (HCZ < −2 SD) were calculated using HIV-unexposed infants as the reference category. Analyses were undertaken using Prism, version 6.0 (GraphPad, La Jolla, California, USA).

### Ethical approvals

Mothers provided written informed consent to join the ZVITAMBO trial, which was approved by the Medical Research Council of Zimbabwe, Medicines Control Authority of Zimbabwe, Johns Hopkins Bloomberg School of Public Health Committee on Human Research and Montreal General Hospital Ethics Committee.

## Results

At baseline, 4495 mothers were HIV-infected and 9561 HIV-uninfected. A total of 405 infants were excluded because of indeterminate/missing maternal HIV status at baseline (*n* = 54) or maternal seroconversion during follow-up (*n* = 351). A total of 13 647 of the remaining infants (96.7% of total study population) had at least one head circumference measurement at a time of known HIV infection/exposure status and were included in the analysis.

### Baseline characteristics

Mothers and infants differed significantly across groups at baseline [1]. Compared with HIV-unexposed infants, HEU and HIV-infected infants were more likely to be shorter and lighter at birth (Omoni *et al.*, unpublished data). Numbers of children per time point in each group are shown in Table 1.

### Mean head circumference-for-age *Z*-scores

**Fig. 1:**
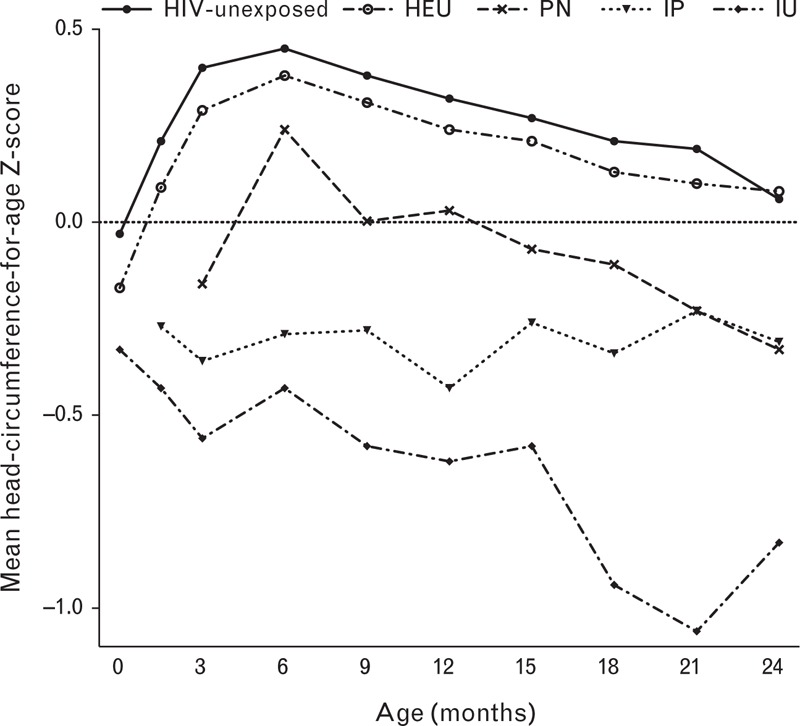
Mean head circumference-for-age *Z*-scores according to child HIV infection/exposure status.

HEU, HIV-exposed uninfected; IP, intrapartum-infected; IU, *in utero*-infected; PN, postnatally-infected.

At birth, mean HCZ was below 0 in IU and HEU children, but not significantly different from 0 in HIV-unexposed children (Table 1). HCZ in HIV-uninfected (HIV-unexposed and HEU) and PN children increased to 6 months of age, followed by a decline to 24 months, a trend not seen in IP and IU children (Fig. 1). As the increase in PN HCZ between 3 and 6 months may have been influenced by the addition of PN infants first testing positive at 6 months, we excluded these infants in a sensitivity analysis; a similar trajectory was seen (data not shown). IP and IU children had significantly lower HCZ than HIV-uninfected children throughout follow-up. PN-infected children had poorer head growth than HIV-uninfected children except at 6 months. HEU children had statistically lower HCZ than HIV-unexposed children in the first year of life.

Among HIV-infected children, IU had the lowest HCZ, which progressively declined throughout follow-up, followed by IP children. During the second year of life, HCZ among PN children declined to reach a similar mean as IP-infected children (Fig. 1). As the PN group may have inadvertently included IP-infected infants testing PCR-negative at 6 weeks of age, two sensitivity analyses were undertaken. Infants first testing HIV-positive at 3 months of age were either grouped with IP infants or excluded from the analysis entirely, with no significant effect on results (data not shown).

### Microcephaly

Microcephaly was more common among IU and IP children than HIV-uninfected children through 24 months. Overall, 11.1% of perinatally infected children had microcephaly at birth, and incidence remained high among IP and IU children throughout follow-up. HEU children had more microcephaly than HIV-unexposed children until 12 months. PN children had a higher incidence of microcephaly than HIV-uninfected children through most of the follow-up period; this reached statistical significance at 9 months of age (Table 1).

## Discussion

The current study has three key findings. First, head growth was poor among HIV-infected Zimbabwean children between birth and 2 years of age; second, head circumference in these children was associated with timing of HIV acquisition; and third, head growth in HEU children was significantly poorer than in HIV-unexposed children during the first year of life.

Overall, 11.1% of HIV-infected infants, compared with 5.4% of HIV-unexposed infants, had microcephaly at birth, highlighting the burden of HIV-associated fetal head growth restriction before ART availability. IU and IP children had lower mean HCZ and more microcephaly than HIV-uninfected children throughout 2 years of follow-up. Compared with HIV-uninfected children, PN children had significantly more microcephaly only at 9 months; the nonsignificant differences at other time points may result from the small number of PN children included in the analysis. Poor head growth in HIV-infected children has previously been reported from Rwanda [12] and the USA [13], and developmental delay among HIV-infected children is well recognized [4]. However, the association between head growth and neurodevelopment is less certain; although there are associations between postnatal head growth and developmental delay in preterm infants [14], there are limited data in the context of HIV [6]. An ART-era Zimbabwean study found no differences in head circumference between HIV-infected and HIV-uninfected infants at birth, but did show an association between microcephaly at birth and neurodevelopmental impairment [6]. In the USA, HIV-infected children had lower developmental scores and head circumferences than HIV-unexposed children at 24 months [15], although associations between development and head circumference were unclear. The impact of microcephaly in the context of HIV in sub-Saharan Africa warrants further investigation. In low-income settings, in which developmental examinations are difficult to access, head circumference could be a helpful screening tool to highlight children who may benefit from formal neurodevelopmental assessment and intervention.

Among HIV-infected children, head growth was associated with timing of HIV acquisition. IU children had particularly poor head growth, with mean HCZ scores that declined throughout the study period. Overall, those infected before birth had lower HCZ than children infected intrapartum or postnatally. This is consistent with previous findings from this cohort [1,3] and other studies [16] showing that IU children have particularly rapid disease progression. However, in contrast to previous findings, toward the end of the study period, IP outcomes were closer to PN than IU outcomes, highlighting the critical influence of intrauterine HIV transmission on head growth. Timing of HIV infection has previously been associated with neurodevelopmental outcomes [17], but children in the trial did not undergo developmental assessments.

Head growth in HEU children was significantly poorer than in HIV-unexposed children through the first year of life; it is plausible that we were underpowered to show significant differences in the second year of life. Similar findings were reported from Colombia, where head circumferences of 1-month-old HEU infants were significantly lower than HIV-unexposed infants, despite being in the normal range [18]. This finding may be specific to HEU infants in low-income and middle-income countries; in the USA, head circumference differences between HEU and HIV-unexposed infants at a mean age of 10 months did not reach statistical significance (HEU mean HCZ 0.18 vs. HIV-unexposed mean HCZ 0.23, *P* = 0.75), although the study may have been underpowered (*n* = 164) [19]. Studies comparing neurodevelopmental outcomes in HEU children have reported heterogeneous results [4]. After adjusting for socioeconomic status, HEU and HIV-unexposed children from Thailand had similar neuroanatomical measurements at 10 years of age [20]. However, HEU Thai children aged 2–12 years had lower IQs than HIV-unexposed controls, even after adjusting for confounders [21].

An important limitation of this study was that very low birthweight infants (<1500 g) were excluded from the original trial, meaning that the most severely growth-restricted infants could not be included in this analysis. Neurodevelopmental assessments were not carried out, meaning that the functional significance of restricted head growth remains uncertain. However, this study has several strengths. First, the HIV status of mothers and children was well characterized throughout follow-up, allowing a detailed insight into the impact of infection timing on head growth. Second, this is the largest HEU infant cohort to date, with a demographically similar HIV-unexposed population. Whether differences in head growth are seen in the current era of preventing mother-to-child transmission and early infant ART is uncertain, but clearly warrants investigation. Although it is plausible that maternal virological suppression and immune reconstitution on ART may reduce the effects of HIV infection on head growth, as shown in one South African study [22], ART may itself adversely affect head growth; one US study showed lower 12-month HCZ in children born to mothers using tenofovir-based compared with non-tenofovir-based regimens [23].

In summary, head circumference in early life was associated with HIV infection/exposure status. The reasons for poor head growth may relate to increased incidence of common infections [3], which may disturb the growth hormone-insulin-like growth factor-1 axis [24]; congenital coinfections, including cytomegalovirus [25]; or HIV itself. Since the first 1000 days (i.e. the period from conception through 2 years of age) is a developmentally sensitive period during which brain growth, neural pathways and synaptic pruning occur, the relationship between head growth and neurodevelopment requires careful evaluation. Future studies should seek to correlate head growth, neurodevelopmental status and neurobiological measures in HIV-infected and HEU infants in developing countries to determine the predictive value of microcephaly for long-term outcomes in these groups of vulnerable children.

## Acknowledgements

C.E. and A.J.P. designed the analysis. C.E., B.C. and R.N. carried out the analysis. C.E. wrote the first draft of the manuscript. B.C., R.N., J.H.H. and A.J.P. critically reviewed and revised the manuscript. J.H.H. designed and recruited to the original trial, and R.N. was a coinvestigator on the original trial.

The ZVITAMBO trial was supported by the Canadian International Development Agency (CIDA) (R/C Project 690/M3688), United States Agency for International Development (USAID) (cooperative agreement number HRN-A-00–97-00015-00 between Johns Hopkins University and the Office of Health and Nutrition – USAID) and a grant from the Bill and Melinda Gates Foundation, Seattle, Washington, USA. Additional funding was received from the SARA Project that is operated by the Academy for Educational Development, Washington, District of Columbia, USA and is funded by USAID's Bureau for Africa, Office of Sustainable Development under the terms of Contract AOT-C-00-99-00237-00, the Rockefeller Foundation (New York, New York, USA) and BASF (Ludwigshafen, Germany). C.E. is funded by the National Institute for Health Research. A.J.P. is funded by the Wellcome Trust (108065/Z/15/Z).

### Conflicts of interest

There are no conflicts of interest.

## Figures and Tables

**Table 1:** Head circumference and microcephaly among children according to HIV infection/exposure status.

	Age at visit									
	Baseline	6 weeks	3 months	6 months	9 months	12 months	15 months	18 months	21 months	24 months
Mean head circumference-for-age Z score; mean (95% confidence interval)										
IU	−0.33 (−0.46, −0.21)^a,b^	−0.43 (−0.59, −0.26)^a,b^	−0.56 (−0.75, −0.38)^a,b^	−0.43 (−0.67, −0.20)^a,b,c^	−0.58 (−0.79, −0.37)^a,b,c,d^	−0.62 (−0.86, 0.39)^a,b,c^	−0.58 (−0.86, −0.31)^a,b,c^	−0.94 (−1.25, −0.64)^a,b,c,d^	−1.06 (−1.46, −0.66)^a,b,c,d^	−0.83 (−1.24, −0.42)^a,b,c,d^
IP	–	−0.27 (−0.38, −0.16)^a,b^	−0.36 (−0.48, −0.23)^a,b^	−0.29 (−0.43, −0.14)^a,b,c^	−0.28 (−0.43, −0.13)^a,b,c^	−0.43 (−0.60, −0.26)^a,b,c^	−0.26 (−0.45, −0.06)^a,b^	−0.34 (−0.56, −0.11)^a,b^	−0.23 (−0.50, 0.04)^a,b^	−0.31 (−0.61, 0.00)^a,b^
PN	–	−	−0.16 (−0.47, 0.15)^a,b^	0.24 (−0.03, 0.51)	0.003 (−0.21, 0.22)^a,b^	0.03 (−0.14, 0.20)^a,b^	−0.07 (−0.25, 0.11)^a,b^	−0.11 (−0.28, 0.07)^a,b^	−0.23 (−0.43, −0.03)^a,b^	−0.33 (−0.53, −0.13)^a,b^
HEU	−0.17 (−0.21, −0.14)^a^	0.09 (0.04, 0.13)^a^	0.29 (0.25, 0.34)^a^	0.38 (0.33, 0.42)^a^	0.31 (0.27, 0.36)^a^	0.24 (0.19, 0.29)^a^	0.21 (0.15, 0.26)	0.13 (0.07, 0.20)	0.10 (0.03, 0.18)	0.08 (0.01, 0.15)
HU	−0.03 (−0.05, 0.01)	0.21 (0.18, 0.24)	0.40 (0.37, 0.42)	0.45 (0.42, 0.48)	0.38 (0.35, 0.40)	0.32 (0.29, 0.35)	0.27 (0.23, 0.32)	0.21 (0.16, 0.26)	0.19 (0.14, 0.25)	0.06 (0.00, 0.12)
Microcephaly; % (*n*)										
IU	11.1 (38)	13.9 (37)	14.5 (30)	11.2 (16)	10.3 (12)	13.5 (14)	13.8 (9)	13.2 (7)	17.1 (7)	15.2 (5)
IP	−	9.4 (47)	10.3 (37)	8.7 (25)	6.8 (16)	11.4 (23)	7.5 (10)	9.8 (10)	7.4 (5)	8.8 (5)
PN	−	−	4.3 (2)	3.9 (3)	5.3 (6)	3.6 (5)	2.5 (3)	1.1 (1)	3.4 (3)	2.5 (2)
HEU	6.8 (272)	4.4 (100)	3.6 (76)	1.4 (31)	1.6 (32)	2.1 (41)	2.3 (30)	2.9 (29)	2.7 (20)	2.3 (16)
HU	5.4 (467)	3.4 (213)	2.4 (156)	1.5 (98)	1.7 (106)	1.9 (121)	1.6 (37)	2.1 (36)	2.4 (30)	2.9 (35)
Odds ratio for microcephaly; mean (95% confidence interval)										
IU	1.86 (1.32, 2.63)^e^	4.54 (3.13, 6.60)^e^	6.78 (4.46, 10.31)^e^	7.58 (4.35, 13.20)^e^	6.59 (3.52, 12.35)^e^	7.84 (4.34, 14.16)^e^	9.65 (4.44, 20.96)^e^	7.17 (3.03–16.96)^e^	8.39 (3.44–20.45)^e^	6.06 (2.21–16.62)^e^
IP	^–^	2.94 (2.11, 4.08)^e^	4.58 (3.15, 6.67)^e^	6.31 (4.00, 9.97)^e^	3.90 (2.27, 6.69)^e^	6.51 (4.07–10.42)^e^	4.84 (2.35, 9.97)^e^	5.12 (2.46–10.64)^e^	3.24 (1.21–8.62)^e^	3.26 (1.22–8.67)^e^
PN	–	–	1.82 (0.44, 7.57)	2.63 (0.82, 8.49)	3.24 (1.39, 7.53)^e^	1.88 (0.76–4.67)	1.57 (0.48, 5.19)	0.50 (0.07–3.70)	1.46 (0.44–4.87)	0.88 (0.21–3.73)
HEU	1.34 (1.15, 1.59)^e^	1.26 (0.98, 1.60)	1.39 (1.05, 1.84)^e^	0.93 (0.62, 1.39)	0.92 (0.62, 1.37)	1.10 (0.77, 1.58)	1.39 (0.86, 2.27)	1.40 (0.85–2.30)	1.08 (0.61–1.92)	0.78 (0.43–1.42)
HU	1.00	1.00	1.00	1.00	1.00	1.00	1.00	1.00	1.00	1.00

*P* < 0.05 for all values compared with unexposed children (unless otherwise stated). *N* at baseline for HIV-unexposed, HEU and IU infants, respectively: 9133, 4029 and 379; *N* at 6 weeks for HIV-unexposed, HEU, IP and IU infants, respectively: 6227, 2350, 499 and 267; *N* at 3 months for HIV-unexposed, HEU, PN, IP and IU infants, respectively: 6399, 2265, 46 and 360, 207; *N* at 6 months for HIV-unexposed, HEU, PN, IP and IU infants, respectively: 6458, 2172, 77, 282 and 143; *N* at 9 months for HIV-unexposed, HEU, PN, IP and IU infants, respectively: 6221, 2038, 113, 237 and 117; *N* at 12 months for HIV-unexposed, HEU, PN, IP and IU infants, respectively: 6217, 1915, 139, 201 and 104; *N* at 15 months for HIV-unexposed, HEU, PN, IP and IU infants, respectively: 2259, 1324, 118, 134 and 65; *N* at 18 months for HIV-unexposed, HEU, PN, IP and IU infants, respectively: 1732, 1004, 95, 102 and 53; *N* at 21 months for HIV-unexposed, HEU, PN, IP and IU infants, respectively: 1253, 753, 87, 68 and 41; *N* at 24 months for HIV-unexposed, HEU, PN, IP and IU infants, respectively: 1222, 710, 79, 57 and 33. CI, confidence interval; HEU, HIV-exposed uninfected; HU, HIV-unexposed; IP, intrapartum-infected; IU, *in utero* infected; PN, postnatally infected. Superscript letters indicate significant difference in mean HCZ between that group as ^a^HIV-unexposed, ^b^HIV-exposed uninfected, ^c^PN or ^d^IP infants (*P* < 0.05).

^e^Odds ratio for microcephaly significantly different from HIV-unexposed infants (*P* < 0.05).
